# Relationship between early‐life nutrition and ages at menarche and first pregnancy, and childbirth rates of young adults: Evidence from APCAPS in India

**DOI:** 10.1111/mcn.12854

**Published:** 2019-06-23

**Authors:** Arindam Nandi, Jere R. Behrman, Maureen M. Black, Sanjay Kinra, Ramanan Laxminarayan

**Affiliations:** ^1^ Center for Disease Dynamics, Economics & Policy, Washington, DC Washington District of Columbia; ^2^ Departments of Economics and Sociology, Population Studies Center University of Pennsylvania Philadelphia Pennsylvania; ^3^ RTI International Research Triangle Park North Carolina; ^4^ Department of Pediatrics University of Maryland School of Medicine Baltimore Maryland; ^5^ Department of Non‐Communicable Disease Epidemiology London School of Hygiene & Tropical Medicine London UK; ^6^ Center for Disease Dynamics, Economics & Policy, New Delhi New Delhi India; ^7^ Princeton Environmental Institute Princeton University Princeton New Jersey

**Keywords:** APCAPS, child development, fertility, fetal origins, India, menarche

## Abstract

India's Integrated Child Development Services (ICDS) provides daily supplementary nutrition and other public health services to women and children. We estimated associations between exposure to early‐childhood ICDS nutrition and adult reproductive outcomes. During 1987–1990, a balanced protein–calorie supplement called “upma”—made from locally available corn–soya ingredients—was rolled out by subdistricts near Hyderabad and offered to pregnant women and children under age 6 years. In a controlled trial, 15 villages received the supplement and 14 did not. We used data from a 2010–2012 resurvey of adults born during the trial (*n* = 715 in intervention and *n* = 645 in control arms). We used propensity score matching methods to estimate the associations between birth in an intervention village and menarcheal age, age at first pregnancy, and fertility of adults. We found that women born in the intervention group during the trial, as compared with the control group, had menarche 0.45 (95% confidence interval [CI: 0.22, 0.68]; *p* < .001) years later and first pregnancy 0.53 (95% CI [0.04, 1.02]; *p* < .05) years later. Married women from the intervention group had menarche 0.36 (95% CI [0.09, 0.64]; *p* < .01) years later, first cohabitation with partner 0.8 (95% CI [0.27, 1.33]; *p* < .01) years later, and first pregnancy 0.53 (95% CI [0.04, 1.02]; *p* < .05) years later than married women in the control group. There was no significant difference between intervention and control group women regarding whether they had at least one childbirth or the total number of children born. The findings were similar when we employed inverse propensity score weighted regression models.

**Key messages**

- Adequate nutrition during the first 2‐3 years of life can have lasting positive impact on health through adulthood.
- The relationship between early-life nutrition and adult reproductive outcomes are incompletely understood, especially in low‐ and middle‐income countries.
- This study found supplementary nutrition in‐utero and during the first 3 years of life to be longitudinally associated with delayed menarche and delayed first pregnancy among Indian women
- The findings have important policy implications for maternal and child health.

**Abbreviations**
APCAPS: Andhra Pradesh Children and Parents Study
CI: confidence interval
ICDS: Integrated Child Development Services
LMICs: low‐ and middle‐income countries
INCAP: Institute of Nutrition for Central America and Panama
PSM: propensity score matching

## 1 INTRODUCTION

Progress on child survival is among the most significant social achievements in India during the 21st century to date. Under‐5 child mortality in India has fallen sharply from 92 per 1,000 live births in 2000 to 39 in 2017 (World Bank, 2017). The rate of decline has accelerated over time, leading to nearly a million more avoided under‐5 deaths during 2005–2015 when compared with 2000–2005 (Fadel et al., 2017). However, rates of undernutrition among Indian children remain high. In 2016, 38%, 21%, and 36% of Indian children under 5 were stunted, wasted, and underweight, respectively (International Institute for Population Sciences [IIPS], 2016). In 48 districts of India—primarily from poorer and more populous states—more than half of under‐5 children were stunted, and in 38 districts, more than half were underweight (IIPS, 2016).

Adequate early‐life nutrition could reduce childhood mortality and morbidity and improve adult height, cardiovascular health, and cognitive, schooling, and economic outcomes (Adair et al., 2013; Alderman, Hoddinott, & Kinsey, 2006; Almond & Currie, 2011; Behrman et al., 2009; Currie & Vogl, 2013; Grantham‐McGregor et al., 2007; Hoddinott et al., 2013b; Hoddinott, Alderman, Behrman, Haddad, & Horton, 2013; Hoddinott, Maluccio, Behrman, Flores, & Martorell, 2008; Maluccio et al., 2009; Martorell, Horta, et al., 2010; Nandi, Ashok, Kinra, Behrman, & Laxminarayan, 2016; Nandi, Behrman, Bhalotra, Deolalikar, & Laxminarayan, 2017; Nandi, Behrman, Kinra, & Laxminarayan, 2018; Nandi, Behrman, & Laxminarayan, 2019; Stein et al., 2013, 2010; Victora et al., 2008). Some of the best evidence linking early‐childhood nutrition and long‐term outcomes in low‐ and middle‐income countries (LMICs) comes from long‐term follow‐ups of early‐childhood nutritional intervention trials. In the Andhra Pradesh Children and Parents Study (APCAPS) in India, exposure to a daily supplementary nutrition in utero and the first 3 years of life was associated with greater adolescent height and improved measures of insulin resistance and arterial stiffness (Kinra et al., 2008). In the Institute of Nutrition for Central America and Panama (INCAP) trial study in Guatemala, exposure in utero and the first 2 years of life to supplementary protein‐enriched nutrition was linked with higher birthweight, height, head circumference, and height‐for‐age and weight‐for‐age *z* scores (Behrman et al., 2009). Adolescents and adults who were exposed to early‐life nutrition in APCAPS and INCAP also had improved schooling attainment, cognitive test scores, and earnings than the comparison groups (Hoddinott et al., 2008; Maluccio et al., 2009; Martorell, Melgar, Maluccio, Stein, & Rivera, 2010; Nandi et al., 2016; 2018; Nandi, Lutter, & Laxminarayan, 2017).

Little is known about how early‐life supplementary nutrition affects the reproductive health of LMIC adults, including ages at menarche, first cohabitation with partner, and first pregnancy, and the rate and number of childbirths. Observational studies in India, Peru, Vietnam, Kenya, Senegal, and Guatemala have found associations of higher birthweights and lower prepubertal body mass with later menarche and greater height for age at 24 months with lower adult fertility (Aurino, Schott, Penny, & Behrman, 2017; Hoddinott, Behrman, et al., 2013b; Khan, Schroeder, Martorell, Haas, & Rivera, 1996; Leenstra et al., 2005; Simondon et al., 1998). Although several studies in high‐income countries have found that later menarche is associated with greater adolescent and adult height, such relationships have not been examined in LMICs (Aurino et al., 2017; Frisch & Revelle, 1970; He & Karlberg, 2001; Zacharias & Rand, 1983).

Menarcheal age is an important public health indicator. Earlier menarche has been frequently linked with growth cessation, earlier marriage and pregnancy, and higher future risk of breast cancer, diabetes, cardiovascular diseases, depression, and mortality (Ibitoye, Choi, Tai, Lee, & Sommer, 2017; Joinson, Heron, Lewis, Croudace, & Araya, 2011; Lakshman et al., 2008; 2009; Sandhu, Ben‐Shlomo, Cole, Holly, & Davey Smith, 2006; Talma et al., 2013; Zacharias & Rand, 1983). In LMICs, growth cessation, and lower adult height are also known to be associated negatively with cognitive and educational outcomes and future earnings (Almond & Currie, 2011; Currie & Vogl, 2013; Hoddinott, Behrman, et al., 2013b; Victora et al., 2008).

Age at marriage, and age at childbirth and rates of childbirth have important implications for maternal and child health, especially among adolescent and young adult mothers. Early marriage and first pregnancy have been linked with higher rates of anaemia and mortality among women, and lower birthweight, gestational age, breastfeeding rates, nutritional status immunization rates, cognitive test scores, and schooling attainment among children (Chari, Heath, Maertens, & Fatima, 2017; Fall et al., 2015; Gibbs, Wendt, Peters, & Hogue, 2012; Montgomery & Lloyd, 1996; Sonneveldt, DeCormier Plosky, & Stover, 2013; Stover & Ross, 2010; Trussell & Pebley, 1984). There is also a widely recognized child quantity–quality trade‐off in LMICs, that is, higher fertility (quantity) is associated with lower human capital investment and worse health and schooling outcomes of each child (quality; Li, Zhang, & Zhu, 2008; Millimet & Wang, 2011; Rosenzweig & Wolpin, 1980).

In this study, we followed up 1,360 adults of age 20–25 years who were born during the original APCAPS nutritional trial. We estimated associations between exposure to supplementary nutrition and adult reproductive outcomes.

## 2 METHODS

### 2.1 APCAPS data

India's Integrated Child Development Services (ICDS) programme is the largest public supplementary nutrition and development programme for women and young children in the world. It provides a daily cooked meal or uncooked take‐home dry meal to pregnant and nursing women, children below the age of 6 years, and adolescent girls. Additional services under the ICDS include preschool education and immunization for children, health check‐ups and referrals, and nutrition and health education (Ministry of Women and Child Development, 2015). The programme is operationalized from Anganwadi centres.

ICDS was introduced in 1975 and rolled out in a phased manner across India starting in the 1980s. During 1987–1990, the National Institute of Nutrition in Hyderabad conducted a nonrandomized controlled trial to examine the effects of supplementary ICDS nutrition during pregnancy on newborn birthweights. Near Hyderabad in the state of Andhra Pradesh, a cluster of villages with a total population of 30,000, was chosen from two adjacent subdistricts—one each for intervention and control groups. In each subdistrict, contiguous villages within a 10‐km radius of a centrally located prominent village (based on the area map) were included, resulting in 15 intervention and 14 control villages.

In the intervention villages, all pregnant women and children below the age of 6 years were offered daily cooked meals at the Anganwadi centres, which they could eat there or take home. The meals—called “upma”—were prepared from locally available corn–soya blends and provided on average 500 kcal of energy and 20–25 g of protein to women and half of those amounts to children (Kinra et al., 2008). In control villages, supplementary nutrition was introduced 3 years after the study ended. Prior to the trial, the intervention and control groups had similar availability of public health services such as an anaemia control in pregnancy, routine childhood vaccination, and primary health care. An abstract describing the trial was published, but no further data—including information on consumption of cooked meals by participants—are available (Kinra et al., 2008).

**Figure 1:**
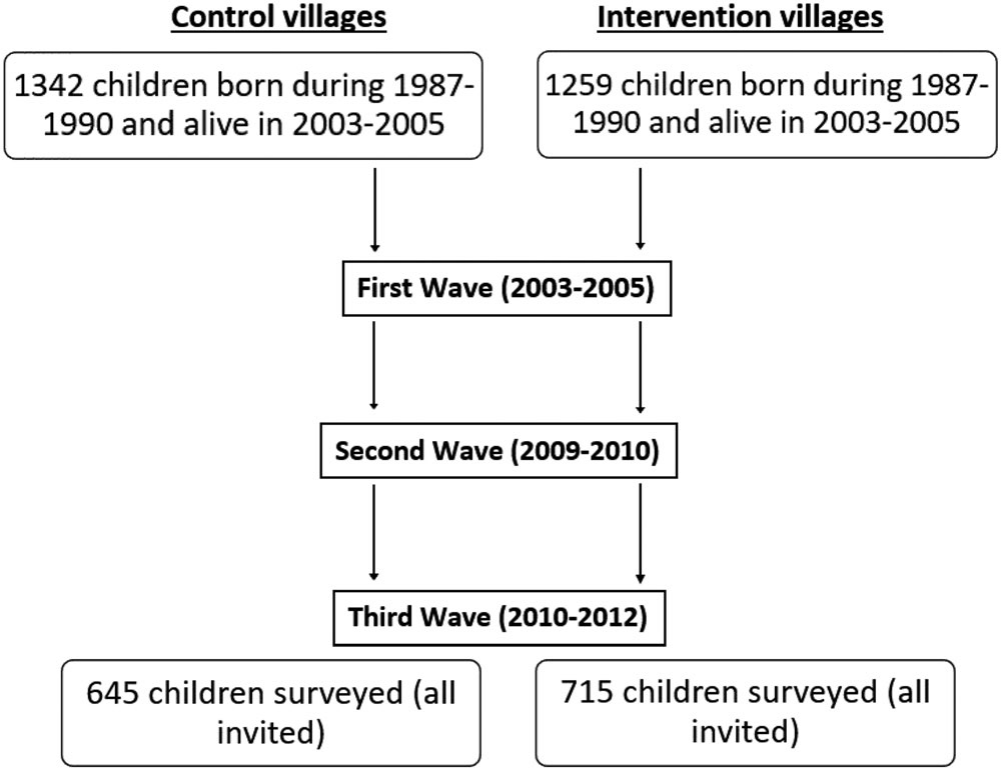
Participants of the APCAPS

The study enrolled all children born between January 1, 1987, and December 31, 1990, in 29 villages. During 2003–2005, a follow‐up study identified 1,342 and 1,259 living children who were born to 1,001 and 962 women, respectively, in intervention and control villages during the original trial period (Kinra et al., 2008). Among them, families who could be matched with baseline records were invited for the survey. Along with their mothers, 654 and 511 children in intervention and control villages participated in the survey that was administered from a clinic in each village. During 2009–2010 and 2010–2012, further follow‐up surveys covered 1,446 and 1,360 children (Figure 1).

Together, the surveys have collected data on a wide range of outcomes including socio‐economic and demographic factors, anthropometry, lifestyle, medical history, cardiovascular physiology, spirometry, and biomarkers (Kinra et al., 2014). The original trial and the follow‐up surveys are collectively known as the APCAPS. The data collection processes have been discussed in previous studies (Kinra et al., 2008, 2014; Kinra, Sarma, Hards, Smith, & Ben‐Shlomo, 2011).

### 2.2 Ethics statement

Ethical clearance for the APCAPS cohort study was provided by the ethics review committees of the National Institute of Nutrition, the Indian Council of Medical Research, the Public Health Foundation of India, the University of Bristol, and the London School of Hygiene and Tropical Medicine. Local administrative authorities in all villages also provided approval, and participants provided informed consent.

### 2.3 Propensity score matching analysis

We used the 2010–2012 wave of the APCAPS survey, in which those who were born during the original trial (hereafter referred to as index adults) were 20–25 years old. We estimated associations between birth in an intervention village and the following reproductive outcomes of index adults: (a) ages at menarche in years for women, (b) ages at first pregnancy in years for women, (c) binary indicator of whether the adults had at least one child, and (d) total number of children (including zero) born to the adults. All outcomes were self‐reported.

As discussed in previous APCAPS studies, there were some statistically significant differences in socio‐economic and demographic characteristics of the intervention and control group adults, which could bias ordinary least square estimates of associations between birth in intervention villages and outcome variables (Kinra et al., 2014; Nandi et al., 2016; 2018). We used a quasi‐experimental propensity score matching (PSM) technique to mitigate such differences (Dehejia & Wahba, 2002; Heckman, Ichimura, & Todd, 1997; Leuven & Sianesi, 2003; Rosenbaum & Rubin, 1984).

First, we conducted a probit regression of birth in intervention villages for index adults on household and individual socio‐economic and demographic characteristics determined before the trial including age in years, sex (whether female), indicators of caste groups (scheduled caste or scheduled tribe, and other backward classes) and religion (non‐Hindu), and parental schooling attainment (literate, completed primary, and completed secondary or above).

Based on the predicted probability from the probit model (known as the propensity score), we matched intervention group index adults with similar index adults from the control group. We restricted our sample to individuals with overlapping propensity scores from the two groups (known as “common support”) and used a Kernel (Epanechnikov) matching algorithm (Caliendo & Kopeinig, 2008; Dehejia & Wahba, 1999; 2002).

Our estimation approach assumes that unobserved characteristics of the intervention and control groups were randomly distributed after matching. Consequently, the average difference in an outcome variable between the intervention and matched control group could be attributed to supplementary ICDS nutrition. The difference is known as the “intent‐to‐treat” estimator.

In addition to the analyses of fertility outcomes of all index adults, we also evaluated outcomes separately for women and ever‐married women. We considered *p* < .05 for statistical significance.

### 2.4 Inverse propensity score weighted regression analysis

We used additional propensity score weighted multivariate regression models to examine associations of the intervention (Hirano & Imbens, 2001; Hirano, Imbens, & Ridder, 2003; McCaffrey, Ridgeway, & Morral, 2004). This technique balances the distribution of covariates between the intervention and control groups and produces statistically efficient estimators. We used the propensity score derived from the first‐stage probit model of PSM as discussed earlier. The weight was defined as the inverse of the propensity score for the intervention group and inverse of one minus the propensity score for the control group (Hirano et al., 2003; Hirano & Imbens, 2001; McCaffrey et al., 2004).

These weights were employed in regression analyses of outcomes. We used Cox proportional hazards models for age at menarche and age at first pregnancy, probit regression for the binary indicator of whether the adult had at least one child, and Poisson regression for total number of children (including zero) born to the adult.

Along with the indicator of birth in intervention villages, the following variables were included as regression covariates: age in years and sex (whether female) when applicable, indicators of caste groups and religion, and parental schooling attainment. Standard errors were robust clustered at the village level.

### 2.5 Tests of matching quality and additional sensitivity analyses

We conducted three tests of matching quality in the base model, that is, whether PSM was successful in reducing differences in characteristics between the intervention and control groups. We computed the standardized percentage bias—which was the difference of the sample means of a covariate between the two groups as a percentage of the square root of the average of the sample variances of the groups—separately before and after matching (Rosenbaum & Rubin, 1985). A successful PSM would reduce the average bias substantially from prematching to postmatching. We also re‐estimated the propensity score regression using the matched intervention–control sample. Under a valid PSM, the pseudo‐*R* ^2^ of this model should be substantially lower as compared with the original propensity score model (Sianesi, 2004). Finally, we conducted a likelihood ratio test of the joint significance of covariates prematching and postmatching. Failure to reject the null hypothesis of joint significance after matching would indicate that the matched sample was homogenous in nature and that the covariates would not adequately explain intervention status any more after matching.

Age at first pregnancy may be correlated with age of marriage (Nandi et al., 2018). Any associations of the intervention with this earlier‐in‐life event could possibly drive its association with ages at first pregnancy. We conducted additional analyses to explore these potential pathways, as discussed in the Supporting Information. We also tested for sensitivity of PSM by examining additional matching algorithms, as presented in the Supporting Information.

## 3 RESULTS

### 3.1 Characteristics of the study sample

Summary statistics of all index men and women are presented in Table 1. In the intervention and control groups, there were 715 and 645 index adults (of them, 272 and 252 women), respectively. There were 160 and 167 married women, respectively, in the two groups, and their summary statistics are presented in Table S5. There was no statistical difference in the proportion of married women between the two groups. Due to missing covariate values, two sample observations were lost for analysis.

**Table 1:** Summary statistics of index adult men and women—APCAPS third wave (2010–2012)

Variable	Index adult men and women			Index adult women		
	Intervention villages	Control villages	Difference	Intervention villages	Control villages	Difference
Age in years	22.9 ± 1.2	22.8 ± 1.2	0.10	22.9 ± 1.3	22.78 ± 1.2	0.16
Whether female, proportion	0.38 ± 0.49	0.39 ± 0.49	−0.01	—	—	—
Literate but no formal education, proportion	0.03 ± 016	0.05 ± 0.21	−0.02*	0.03 ± 0.18	0.06 ± 0.23	−0.02
Completed primary education, proportion	0.12 ± 0.32	0.15 ± 0.36	−0.03	0.15 ± 0.35	0.18 ± 0.39	−0.04
Completed secondary education or higher, proportion	0.83 ± 0.38	0.74 ± 0.44	0.09**	0.78 ± 0.42	0.67 ± 0.47	0.11**
Employed or enrolled in higher education, proportion	0.72 ± 0.45	0.65 ± 0.48	0.07**	0.39 ± 0.49	0.35 ± 0.48	0.04
Ever married by age 20–25 years, proportion	0.29 ± 0.45	0.35 ± 0.48	−0.06*	0.59 ± 0.49	0.66 ± 0.47	−0.07
Whether had at least one child, proportion	0.19 ± 0.39	0.23 ± 0.42	−0.04	0.43 ± 0.5	0.48 ± 0.5	−0.05
Total number of children born	0.29 ± 0.67	0.38 ± 0.76	−0.08*	0.69 ± 0.91	0.82 ± 0.98	−0.13
Menarcheal ages in years	—	—	—	12.96 ± 1.37	12.63 ± 1.14	0.33**
Ages at first pregnancy in years	—	—	—	19.31 ± 1.86	18.82 ± 2.16	0.49*
Scheduled caste/scheduled tribe, proportion	0.43 ± 0.49	0.32 ± 0.47	0.11***	0.47 ± 0.5	0.33 ± 0.47	0.14***
Other backward classes, proportion	0.48 ± 0.5	0.61 ± 0.49	−0.13**	0.44 ± 0.5	0.58 ± 0.5	−0.13**
Non‐Hindu household, proportion	0.04 ± 0.19	0.08 ± 0.27	−0.04**	0.03 ± 0.18	0.11 ± 0.31	−0.07**
Wealth quintile 1, proportion	0.18 ± 0.39	0.22 ± 0.41	−0.04	0.2 ± 0.4	0.24 ± 0.43	−0.04
Wealth quintile 2, proportion	0.23 ± 0.42	0.17 ± 0.37	0.06**	0.24 ± 0.43	0.18 ± 0.39	0.06
Wealth quintile 3, proportion	0.21 ± 0.41	0.19 ± 0.39	0.03	0.2 ± 0.4	0.15 ± 0.36	0.05
Wealth quintile 4, proportion	0.21 ± 0.41	0.19 ± 0.39	0.03	0.21 ± 0.41	0.18 ± 0.38	0.04
Wealth quintile 5, proportion	0.16 ± 0.37	0.24 ± 0.43	−0.07**	0.14 ± 0.35	0.26 ± 0.44	−0.11**
Father literate, proportion	0.1 ± 0.31	0.11 ± 0.31	0.00	0.11 ± 0.32	0.11 ± 0.32	0.00
Father's education: primary, proportion	0.13 ± 0.34	0.16 ± 0.37	−0.03	0.13 ± 0.34	0.2 ± 0.4	−0.07*
Father's education: secondary and above, proportion	0.08 ± 0.27	0.09 ± 0.29	−0.02	0.06 ± 0.24	0.12 ± 0.33	−0.06*
Mother literate, proportion	0.12 ± 0.33	0.15 ± 0.36	−0.03	0.13 ± 0.33	0.21 ± 0.41	−0.08*
*n*	715	644		272	252	

*Note*. Values are mean ± standard deviation, unless stated otherwise. Difference is between the mean values of intervention and control groups. Index adults are those born in study villages during the original trial period of 1987 to 1990 and alive at the time of the survey. Wald *t* tests for continuous variables and *z* tests for proportions were used to examine the statistical significance of the differences between intervention and control group means.

* *p* < .05.

** *p* < .01.

*** *p* < .001.

Intervention village adults had completed secondary education and were employed or enrolled in higher education at significantly higher proportions. Lower proportions of them were ever married, and they had fewer children than control group adults. Intervention group women had higher menarcheal and first pregnancy ages as compared with control group women. The proportion of intervention group households belonging to scheduled caste or scheduled tribe groups was higher compared with the control group, whereas those belonging to other backward classes was lower. Higher proportions of intervention group households belonged to the middle three wealth quintiles as compared with the control group.

### 3.2 Propensity score matching estimates

The PSM intent‐to‐treat estimators of associations between birth in an intervention village and adult reproductive outcomes are presented in Table 2.

**Table 2:** Propensity score matching estimates of associations between birth in an intervention village and adult reproductive outcomes

Sample		Ages at menarche	Ages at first pregnancies	Whether had at least one child	Total number of children born
	*n*	Estimate [95% CI]	Estimate [95% CI]	Estimate [95% CI]	Estimate [95% CI]
Index men and women	1,358	NA	NA	−0.05 [−0.09, 0]*	−0.1 [−0.18, −0.02]*
Index women	518	0.45 [0.22, 0.68]***	0.53 [0.04, 1.02]*	−0.1 [−0.19, −0.01]*	−0.22 [−0.4, −0.05]*
Index men	835	NA	NA	−0.03 [−0.06, 0]	−0.05 [−0.09, 0]*
Married index men and married index women	427	NA	NA	−0.01 [−0.1, 0.08]	−0.09 [−0.27, 0.09]
Married index women	326	0.36 [0.09, 0.64]**	0.53 [0.04, 1.02]*	0.01 [−0.09, 0.11]	−0.1 [−0.31, 0.11]
Married index men	101	NA	NA	−0.02 [−0.23, 0.19]	−0.08 [−0.38, 0.21]

*Note*. Values are propensity score matching estimates of associations between birth in an intervention village and outcome variables, along with 95% CIs. Kernel (Epanechnikov) matching algorithm was used.

Abbreviation: CI, confidence interval.

* *p* < .05.

** *p* < .01.

*** *p* < .001.

Ages at menarche and first pregnancy were 0.45 (95% confidence interval [CI: 0.22, 0.68]; *p* < .001) and 0.53 (95% CI [0.04, 1.02]; *p* < .05) years higher, respectively, among intervention village index adult women, compared with matched control group index adult women. The proportion of intervention group women having at least one child was 0.1 (95% CI [−0.19, −0.01]; *p* < .05) lower, and 0.22 (95% CI [−0.4, −0.05]; *p* < .05) fewer children were born to them, compared with matched control group women.

Among married index women, ages at menarche and first pregnancy were higher by 0.36 (95% CI [0.09, 0.64]; *p* < .01) and 0.53 (95% CI [0.04, 1.02]; *p* < .05) years, respectively, among the intervention group, compared with the control group.

In the full sample of index men and women, the proportion of intervention village adults having at least one child was 0.05 (95% CI [−0.09, 0]; *p* < .05) lower, and they had −0.1 (95% CI [−0.18, −0.02]; *p* < .05) fewer children than matched control adults. Intervention group index men also had 0.05 (95% CI [−0.09, 0]; *p* < .05) fewer children as compared with matched control men.

### 3.3 Results from inverse propensity score weighted regression

Results of weighted regression analysis are presented in Table 3. Menarcheal ages were higher among intervention village women and married women—with estimated hazard ratios of menarche onset of 0.77 (95% CI [0.67, 0.88]; *p* < .001) and 0.79 (95% CI [0.68, 0.92]; *p* < .01), respectively—as compared with the control groups.

**Table 3:** Regression‐based estimates of associations between birth in an intervention village and adult reproductive outcomes, using inverse propensity score weights

Sample		Ages at menarche	Ages at first pregnancies	Whether had at least one child	Total number of children born
	*n*	Estimate [95% CI]	Estimate [95% CI]	Estimate [95% CI]	Estimate [95% CI]
Index men and women	1358	NA	NA	−0.05 (−0.09, −0.01)*	−0.3 (−0.53, −0.07)*
Index women	523	0.77 (0.67, 0.88)***	0.88 (0.71, 1.08)	−0.08 (−0.18, 0.01)	−0.26 (−0.48, −0.03)*
Index men	835	NA	NA	−0.02 (−0.05, 0.01)	−0.56 (−1.37, 0.26)
Married index men and married index women	427	NA	NA	−0.02 (−0.11, 0.07)	−0.11 (−0.25, 0.03)
Married index women	326	0.79 (0.68, 0.92)**	0.88 (0.71, 1.08)	0 (−0.1, 0.1)	−0.08 (−0.22, 0.06)
Married index men	101	NA	NA	−0.07 (−0.28, 0.14)	−0.3 (−0.8, 0.2)

*Note*. Values are the estimated coefficient of the indicator of birth in an intervention village in the regression of the outcome variable, along with 95% CIs, unless stated otherwise. Estimated coefficients of other regression covariates are not shown. We used Cox proportional hazards model for age at menarche and age at first pregnancy, marginal effect probit regression models for whether the adult had at least one childbirth, and Poisson regression models for the number of children born. For Cox models, hazard ratios are shown instead of coefficients. All regression models were weighted using inverse propensity score weights. Standard errors were clustered at the village level.

Abbreviation: CI, confidence interval.

* *p* < .05.

** *p* < .01.

*** *p* < .001.

In the full sample of index men and women, the proportion of intervention village adults with at least one child was 0.05 (95% CI [−0.09, −0.01]; *p* < .05) lower, and they had 0.3 (95% CI [−0.53, −0.07]; *p* < .05) fewer children, as compared with control village adults. Intervention village index women had 0.26 (95% CI [−0.48, −0.03]; *p* < .05) fewer children as compared with control group women.

### 3.4 Results from tests of matching quality

As shown in Table 4, PSM successfully reduced differences in the characteristics of the intervention and control groups. The mean standardized percentage bias, that is, average difference between the two groups in the covariates of the first stage regression, reduced by 77–90% from prematching to postmatching across all models. Only in the subsample of married index men, the reduction was smaller, likely due to low sample size. Detailed results of these balance tests for each covariate are presented in Tables S6 and S7.

**Table 4:** Summary of tests of matching quality

Analysis of index adults	Mean % bias in unmatched data	Mean % bias after PSM	Pseudo‐*R* ^2^ before matching	Pseudo‐*R* ^2^ after matching	*p*‐value for *χ* ^2^ before matching	*p*‐value for *χ* ^2^ after matching
Index men and women	11.3	2.3	.02	.002	0	.92
Index women	11.3	1.7	.02	.001	0	1
Index men	11.3	2.6	.02	.002	0	.98
Married index men and married index women	11.3	1.7	.02	.001	0	1
Married index women	11.3	1.2	.02	.001	0	1
Married index men	11.3	8.2	.02	.012	0	.99

*Note*. Standardized percentage bias was measured as the difference of the sample means of a covariate between the two groups as a percentage of the square root of the average of the sample variances of the groups. Matching was based on propensity scores, using the Kernel (Epanechnikov) matching method.

Abbreviation: PCM, propensity score matching.

Similarly, the value of pseudo‐*R* ^2^ reduced by more than 90% from prematching to postmatching, and the likelihood ratio test statistic of joint significance of the covariates became statistically significant to nonsignificant from prematching to postmatching. These results show that the PSM method was valid.

## 4 DISCUSSION

Using PSM and weighted regression analysis, we observed that 20‐ to 25‐year‐old women who were exposed to supplementary nutrition in utero and during the first 3 years of life under a controlled trial had menarche and first cohabitation with a partner at older ages as compared with observationally identical women who were not exposed to the supplementary nutrition. Intervention group women (and men) had fewer childbirths compared with matched control group women (and men), although no difference was seen among married women (and men). Because there were no childbirths reported by unmarried adults, differences in childbirths between intervention and control groups were explained fully by lower proportions of marriage in the intervention group (Nandi et al., 2018). In PSM analysis, but not in weighted regressions, intervention group women were also observed to have later first pregnancies as compared with similar control group women. The results were not sensitive to matching algorithms.

These findings contribute substantially to evidence on reproductive and demographic effects of early‐childhood nutrition in LMICs (Almond & Currie, 2011; Behrman & Rosenzweig, 2004; Currie & Vogl, 2013; Victora et al., 2008). To the best of our knowledge, only two studies provide high‐quality, experimental evidence over long time spans. The INCAP study in Guatemala has linked nutrition with improved adult schooling attainment, cognitive test scores, and wages, and among women, higher ages at first pregnancy and fewer pregnancies (Behrman, 2009; Freeman, Klein, Townsend, & Lechtig, 1980; Hoddinott et al., 2013c; Maluccio et al., 2009). Previous APCAPS studies have associated exposure to early‐life nutritional supplementation with improved adolescent heights, cardiovascular risk profiles, and school enrolment and grades, and lower marriage rates (Kinra et al., 2008; Kulkarni et al., 2014; Nandi et al., 2016; 2018).

The relationships between nutritional status and menarcheal ages are complex and are influenced by biological and environmental factors (Aurino et al., 2017; Juul, Chang, Brar, & Parekh, 2017; Pathak, Tripathi, & Subramanian, 2014; Soliman, De Sanctis, & Elalaily, 2014). Whereas genetics are primary determinants, menarcheal ages are also associated positively with maternal well‐being, breastfeeding rates, certain diseases (e.g., diabetes), and negatively with intake of animal proteins, psychological stress, standard of living, and environmental exposure to endocrine disruptors (Abreu & Kaiser, 2016; Aurino et al., 2017; Yermachenko & Dvornyk, 2014). Among anthropometric indicators, lower birthweights and higher prepubertal body masses have been linked with earlier menarche (Adair, 2001; Aurino et al., 2017; Blell, Pollard, & Pearce, 2008; Cooper, Kuh, Egger, Wadsworth, & Barker, 1996; Hui, Leung, Wong, Lam, & Schooling, 2012; Khan et al., 1996; Leenstra et al., 2005; Marcovecchio & Chiarelli, 2013; Persson et al., 1999; Simondon et al., 1998; Sloboda, Hart, Doherty, Pennell, & Hickey, 2007; Tam, de Zegher, Garnett, Baur, & Cowell, 2006). A recent systematic review study indicates that the evidence on the relationship of menarcheal age with prenatal nutrition, infant feeding, and childhood diet patterns remains inconsistent across the world (Villamor & Jansen, 2016).

The determinants of menarcheal age in LMICs have not been widely studied. A recent study using longitudinal Young Lives data on adolescents in India, Peru, and Vietnam found positive associations of birthweight and negative associations of prepubertal height and body mass measures with menarcheal ages (Aurino et al., 2017). Prepubertal stunting was linked with later menarche also in Guatemala, Senegal, and Bangladesh (Bosch, Willekens, Baqui, Van Ginneken, & Hutter, 2008; Khan et al., 1996; Simondon et al., 1998).

In our study, exposure to early‐life nutrition was associated with later menarche and higher variances in menarcheal age. Due to lack of data on birthweight or prepubertal body mass of study participants, we could not examine their relationships with menarcheal age. However, our findings align with previous studies linking earlier puberty with earlier cessation of growth in adolescents (Sandhu et al., 2006; Zacharias & Rand, 1983). The average menarcheal age in our study was 12.8 years, similar to the average in Andhra Pradesh (95% CI [12.7, 13.1 years]) at that time. Then at ages 13–18 years, intervention group participants were 1.4 cm taller (Kinra et al., 2008), possibly due to earlier menarche and growth cessation in the control group. A study of 1,520 men in the United Kingdom found that adolescents with later puberty were 0.6 cm taller as compared with those with earlier puberty (Sandhu et al., 2006). Another study of 286,000 adult women from 10 European countries associated a 1‐year later menarche with 0.3‐cm growth (Onland‐Moret et al., 2005).

Soy protein—an ingredient of the meals offered to the intervention group—may have also affected menarcheal age in our study. Previous studies of adolescents in Germany and the United States have found that higher levels of vegetable protein intake, as compared with lower intake, could delay menarche (Berkey, Gardner, Frazier, & Colditz, 2000; Günther, Karaolis‐Danckert, Kroke, Remer, & Buyken, 2010; Kissinger & Sanchez, 1987). Naturally occurring endocrine disruptors in soy could also delay puberty in girls, although the evidence is not conclusive (Fisher & Eugster, 2014).

The association between early‐life nutrition and age at first pregnancy could be mediated by schooling attainment and age at marriage. Women who completed schooling earlier than other women may be more likely to get married and in turn more likely to become pregnant earlier. In a previous study of the APCAPS adult cohort, we found that higher proportions of intervention group women completed secondary‐ and graduate‐level schooling and lower proportions were married, as compared the control group (Nandi et al., 2018). In the current study, ages at first cohabitation were higher in the intervention group than in the matched control group. This indicates that the pathway of positive association between nutrition and first pregnancy age may be through later cohabitation in the intervention group. In the INCAP study, a one standard deviation increase in height‐for‐age *z* scores at age 2 years was associated with 0.77 years higher ages at first childbirth, 0.63 fewer pregnancies, and 0.43 fewer childbirths among 25‐ to 42‐year‐old women (Hoddinott, Behrman, et al., 2013c). No associations were seen between height‐for‐age *z* scores and age at first marriage.

Our findings have important policy implications. The importance in LMICs of adult height as an indicator of past nutrition and as a determinant of adult health status, cognitive ability, schooling attainment, and economic productivity is well accepted (Almond & Currie, 2011; Currie & Vogl, 2013; Victora et al., 2008). Therefore, supplementary nutrition, which may delay menarche, could potentially improve adult height and cognitive, schooling, and economic outcomes (Aurino et al., 2017).

Ages at first cohabitation and first pregnancy have important implications for maternal and child health and well‐being (Gibbs et al., 2012; Yu, Mason, Crum, Cappa, & Hotchkiss, 2016). In India, a 1‐year delay in marriage for women is associated with lower home birth and higher breastfeeding rates and improved vaccination rates, weight‐for‐height, school enrolment, and cognitive test scores of children (Chari et al., 2017). In a study of five LMICs including India, children of mothers below the age of 19 years were more likely to be born preterm, had lower birthweights and higher rates of 2‐year stunting, and were less likely to complete secondary schooling than children of 20‐ to 24‐year‐old mothers (Fall et al., 2015). In our data, 43% and 38% of married index women started cohabiting with partners and had their first children, respectively, before the age of 19 years. At the national level, 27% of 20‐ to 24‐year‐old Indian women were married before the age of 18, and 8% of 15‐ to 19‐year‐old women in 2016 had pregnancies or childbirths (IIPS, 2016), possibly leading to substantial adverse health effects for their children.

There are limitations to our analysis. Access to public health services such as anaemia control in pregnancy and routine child immunization was similar across intervention and control villages before the trial. Integration of these services though the introduction of daily meals may have increased their use in the intervention villages, on which no data are available. However, such services may increase effects of nutrition by improving overall health of index children and their mothers.

Reproductive outcomes in our study were self‐reported by participants. Data on age at menarche were collected in adolescence, limiting measurement error arising from a long recall period. Data were collected by female surveyors only to limit reporting bias. The average age at menarche in our study was consistent with other concurrent sources from the state (Pathak et al., 2014). Age at first pregnancy and fertility are less likely to be affected by the duration of recall period. Whereas random error, which could underestimate estimated associations, cannot be entirely ruled out, systematic (recall) bias is unlikely as the study participants were unaware of these secondary hypotheses at the time of data collection.

No information was available on which study participants actually consumed the meals offered to them during the trial. Any unknown redistribution of meals within or across households might have weakened the estimated associations with reproductive outcomes in our study. There was also no information on future marriage and pregnancies of unmarried women, requiring us to deal with censored data.

ICDS supplementary nutrition was introduced in control villages after the trial ended. Index adults in these villages were exposed to nutrition during 3–6 years of their lives, potentially weakening the estimated nutritional associations in our study. However, nutrition during the first 2–3 years of life is known to have the largest effect on most future outcomes (Black et al., 2013; Grantham‐McGregor et al., 2007).

Exposure to endocrine disruptors such as pesticides and phthalates have been linked with early puberty, whereas heavy metals have been associated with later puberty (Fisher & Eugster, 2014; Gladen, Ragan, & Rogan, 2000; Ouyang et al., 2005; Özen & Darcan, 2011; Soliman et al., 2014). Such disruptors are widely present in everyday objects and home and outdoor environments (Soliman et al., 2014), and living in an urbanizing, high‐traffic, or polluted environment may result in even greater exposure (McGuinn et al., 2016). Over time, these environmental factors may have evolved differently across the intervention and control villages. Although a 2013 household survey indicated that all study villages were experiencing rapid urbanization and economic development, no further environmental data—especially from the prepubertal periods of women—are available (London School of Hygiene & Tropical Medicine, 2018).

## 5 CONCLUSION

Exposure to supplementary nutrition in utero and during the first 3 years of life was associated with higher ages at menarche, first cohabitation with partners, and first pregnancies among Indian women.

## CONFLICTS OF INTEREST

The authors declare that they have no conflicts of interest.

## CONTRIBUTIONS

SK and other collaborators at APCAPS collected the data. AN conducted the analysis and wrote the first version of the manuscript. JRB, SK, MMB, and RL interpreted the findings and edited the manuscript. AN, RL, and JRB were responsible for the final contents. All authors have read and approved the final manuscript.

## Supporting information

Table S1. Summary statistics of married index adult women – APCAPS third wave (2010–2012)
Table S2. Covariate balance before and after propensity score matching
Table S3: Covariate balance before and after propensity score matching
Table S4: Estimated PSM associations between birth in an intervention village and adult reproductive outcomes – Kernel matching with logit model
Table S5: Estimated PSM associations between birth in an intervention village and adult reproductive outcomes – radius matching
Table S6: Estimated PSM associations between birth in an intervention village and adult reproductive outcomes – one‐to‐one nearest‐neighbour matching
