# Supplementary material for: Relationship between early‐life nutrition and ages at menarche and first pregnancy, and childbirth rates of young adults: Evidence from APCAPS in India
Source: Matern Child Nutr. 2019 Jun 23;16(1):e12854. doi: 10.1111/mcn.12854 (PMC7038893; doi:10.1111/mcn.12854)
Supplement: Supplementary file 1 — Table S1. Summary statistics of married index adult women – APCAPS third wave (2010–2012) Table S2. Covariate balance before and after propensity score matching Table S3: Covariate balance before and after propensity score matching Table S4: Estimated PSM associations between birth in an intervention village and adult reproductive outcomes – Kernel matching with logit model Table S5: Estimated PSM associations between birth in an intervention village and adult reproductive outcomes – radius matching Table S6: Estimated PSM associations between birth in an intervention village and adult reproductive outcomes – one‐to‐one nearest‐neighbour matching [file MCN-16-e12854-s001.docx]

**Supplementary Web Appendix**

**Part A: Pathways of association between intervention and age at first pregnancy**

Age at first pregnancy may be correlated with age of marriage (Nandi et al., 2018). Any associations of the intervention with this earlier-in-life events could possibly drive its association with ages at first pregnancy.

In order to explore this pathway, we examined associations between the intervention and ages at first cohabitation for married women. Using PSM (Kernel matching), age at first cohabitation was 0.8 (95% CI: 0.27, 1.33; p<0.01) years higher among married intervention-village women as compared with matched married women from the control group. Using inverse propensity-score weighted Cox regression model, the estimated hazard ratio for birth in an intervention village was 0.83 (95% CI: 0.7, 0.98; p<0.05), also showing delayed cohabitation among intervention group women as compared with the control group.

**Part B: Additional propensity score matching algorithms**

We used three additional matching algorithms to test the sensitivity of the base PSM model. We replaced the first-stage probit regression with a logit model, also used with Kernel matching. In other models, we used the first-stage probit regression but matched intervention-group observations with similar control-group counterparts within a probability radius of 0.05, and used a one-to-one nearest-neighbor matching method without replacement (Caliendo and Kopeinig, 2008; Dehejia and Wahba, 2002, 1999). Results from these additional matching algorithms in the base model, presented in Supplemental Tables 8 through 10, were similar. Intervention-group index adult women had significantly higher menarcheal and first-pregnancy ages than their control group counterparts. They also had lower rates and number of childbirths, except that there were no statistically significant differences in the subsample of married women.

Supplementary Table 5: Summary statistics of married index adult women – APCAPS third wave (2010-2012)

|  | Intervention villages | Control  villages | Differences in means (intervention – control) | *P*-value of differences |
| --- | --- | --- | --- | --- |
| Age in years | 23.26 ± 1.23 | 23.04 ± 1.14 | 0.23 | 0.08 |
| Literate but no formal education, proportion | 0.05 ± 0.22 | 0.08 ± 0.28 | -0.03 | 0.22 |
| Completed primary education, proportion | 0.19 ± 0.4 | 0.24 ± 0.43 | -0.05 | 0.32 |
| Completed secondary education or higher, proportion | 0.7 ± 0.46 | 0.54 ± 0.5 | 0.16 | 0.00 |
| Employed or enrolled in higher education, proportion | 0.22 ± 0.41 | 0.27 ± 0.45 | -0.05 | 0.29 |
| Whether had at least one child, proportion | 0.73 ± 0.44 | 0.73 ± 0.45 | 0.00 | 0.99 |
| Total number of children born | 1.18 ± 0.91 | 1.24 ± 0.96 | -0.06 | 0.53 |
| Menarcheal ages in years | 12.9 ± 1.34 | 12.6 ± 1.08 | 0.30 | 0.03 |
| Ages at first pregnancy in years | 19.31 ± 1.86 | 18.82 ± 2.16 | 0.49 | 0.04 |
| Scheduled caste/ scheduled tribe, proportion | 0.43 ± 0.5 | 0.37 ± 0.48 | 0.06 | 0.27 |
| Other backward classes, proportion | 0.52 ± 0.5 | 0.57 ± 0.5 | -0.06 | 0.31 |
| Non-Hindu household, proportion | 0.03 ± 0.17 | 0.06 ± 0.24 | -0.03 | 0.21 |
| Wealth quintile 1, proportion | 0.2 ± 0.4 | 0.29 ± 0.46 | -0.09 | 0.06 |
| Wealth quintile 2, proportion | 0.22 ± 0.41 | 0.16 ± 0.37 | 0.06 | 0.2 |
| Wealth quintile 3, proportion | 0.2 ± 0.4 | 0.12 ± 0.32 | 0.08 | 0.04 |
| Wealth quintile 4, proportion | 0.2 ± 0.4 | 0.18 ± 0.39 | 0.02 | 0.68 |
| Wealth quintile 5, proportion | 0.18 ± 0.39 | 0.25 ± 0.43 | -0.07 | 0.14 |
| Father literate, proportion | 0.09 ± 0.29 | 0.11 ± 0.31 | -0.01 | 0.66 |
| Father’s education: primary, proportion | 0.09 ± 0.29 | 0.14 ± 0.35 | -0.05 | 0.16 |
| Father’s education: secondary and above, proportion | 0.04 ± 0.19 | 0.09 ± 0.29 | -0.05 | 0.05 |
| Mother literate, proportion | 0.09 ± 0.28 | 0.18 ± 0.39 | -0.09 | 0.02 |
| *n* | 160 | 167 |  |  |

Note: Values are *mean* $\pm$ *standard deviation*, unless stated otherwise. Index adults are those born in study villages during the original trial period of 1987 to 1990. Wald t-tests for continuous variables and z-tests for proportions were used to examine the statistical significance of the differences between intervention and control group means.

Supplementary Table 6: Covariate balance before and after propensity score matching

|  | % bias (balance) before matching | % bias (balance) after matching |
| --- | --- | --- |
| *Analysis of index men and women:* |  |  |
| Ages in years | 8 | 2.9 |
| Scheduled caste/ scheduled tribe, proportion | 23*** | -0.9 |
| Other backward classes, proportion | -26.8*** | -3.2 |
| Non-Hindu household, proportion | -15.8** | 0.1 |
| Father literate, proportion | -1.8 | -0.2 |
| Father’s education: primary, proportion | -9 | 3.6 |
| Father’s education: secondary and above, proportion | -6.4 | 2.4 |
| Mother literate, proportion | -8.4 | 6.6 |
|  |  |  |
| *Analysis of index women:* |  |  |
| Ages in years | 8 | 0.1 |
| Scheduled caste/ scheduled tribe, proportion | 23*** | 1.2 |
| Other backward classes, proportion | -26.8*** | -3.6 |
| Non-Hindu household, proportion | -15.8** | 2.6 |
| Father literate, proportion | -1.8 | -3.7 |
| Father’s education: primary, proportion | -9 | -1.8 |
| Father’s education: secondary and above, proportion | -6.4 | 1.7 |
| Mother literate, proportion | -8.4 | 0.9 |
|  |  |  |
| *Analysis of index men:* |  |  |
| Ages in years | 8 | 4.5 |
| Scheduled caste/ scheduled tribe, proportion | 23*** | -3.3 |
| Other backward classes, proportion | -26.8*** | 0.1 |
| Non-Hindu household, proportion | -15.8** | 0.5 |
| Father literate, proportion | -1.8 | 3.4 |
| Father’s education: primary, proportion | -9 | 2.5 |
| Father’s education: secondary and above, proportion | -6.4 | 5.3 |
| Mother literate, proportion | -8.4 | 3.9 |

Note: Values are %, unless stated otherwise. Standardized percentage bias was measured as the difference of the sample means of a covariate between the two groups as a percentage of the square root of the average of the sample variances of the groups. Matching was on propensity scores, using a Kernel (Epanechnikov) algorithm. * *P* <0.05, ** *P* <0.01, *** *P* <0.001

Supplementary Table 7: Covariate balance before and after propensity score matching

|  | % bias (balance) before matching | % bias (balance) after matching |
| --- | --- | --- |
| *Analysis of married index men and women:* |  |  |
| Ages in years | 8 | 1.7 |
| Scheduled caste/ scheduled tribe, proportion | 23*** | 1.4 |
| Other backward classes, proportion | -26.8*** | -1.2 |
| Non-Hindu household, proportion | -15.8** | -0.3 |
| Father literate, proportion | -1.8 | -5.1 |
| Father’s education: primary, proportion | -9 | 1 |
| Father’s education: secondary and above, proportion | -6.4 | 0.4 |
| Mother literate, proportion | -8.4 | -0.4 |
|  |  |  |
| *Analysis of married index women:* |  |  |
| Ages in years | 8 | 5.3 |
| Scheduled caste/ scheduled tribe, proportion | 23*** | 1 |
| Other backward classes, proportion | -26.8*** | -0.1 |
| Non-Hindu household, proportion | -15.8** | 1 |
| Father literate, proportion | -1.8 | -2.1 |
| Father’s education: primary, proportion | -9 | 0.1 |
| Father’s education: secondary and above, proportion | -6.4 | 0.9 |
| Mother literate, proportion | -8.4 | -0.7 |
|  |  |  |
| *Analysis of married index men:* |  |  |
| Ages in years | -1 | 8 |
| Scheduled caste/ scheduled tribe, proportion | 7.7 | 23*** |
| Other backward classes, proportion | -1.9 | -26.8*** |
| Non-Hindu household, proportion | -15.8 | -15.8** |
| Father literate, proportion | 19.2 | -1.8 |
| Father’s education: primary, proportion | -2.2 | -9 |
| Father’s education: secondary and above, proportion | -15.7 | -6.4 |
| Mother literate, proportion | -10.3 | -8.4 |

Note: Values are %, unless stated otherwise. Standardized percentage bias was measured as the difference of the sample means of a covariate between the two groups as a percentage of the square root of the average of the sample variances of the groups. Matching was on propensity scores, using a Kernel (Epanechnikov) algorithm. * *P* <0.05, ** *P* <0.01, *** *P* <0.001

Supplemental Table 8: Estimated PSM associations between birth in an intervention village and adult reproductive outcomes – Kernel matching with logit model

|  |  | Ages at menarche | Ages at first pregnancies | Whether had at least one child | Total number of children born |
| --- | --- | --- | --- | --- | --- |
|  | n | Estimate  (95% CI) | Estimate  (95% CI) | Estimate  (95% CI) | Estimate  (95% CI) |
| Index men and women | 1,358 | NA | NA | -0.05 (-0.09, 0)* | -0.09 (-0.17, -0.01)* |
| Index women | 518 | 0.45 (0.22, 0.68)*** | 0.53 (0.04, 1.02)* | -0.1 (-0.19, -0.01)* | -0.25 (-0.43, -0.07)** |
| Index men | 835 | NA | NA | -0.03 (-0.06, 0) | -0.04 (-0.09, 0)* |
| Married index men and married index women | 427 | NA | NA | -0.01 (-0.1, 0.08) | -0.12 (-0.3, 0.07) |
| Married index women | 326 | 0.36 (0.09, 0.64)** | 0.53 (0.04, 1.02)* | -0.01 (-0.11, 0.09) | -0.14 (-0.36, 0.08) |
| Married index men | 101 | NA | NA | 0.01 (-0.09, 0.11) | -0.02 (-0.24, 0.19) |

Note: Values are propensity score matching estimates of associations between birth in an intervention village and outcome variables, along with 95% confidence intervals (CI). Matching was on propensity scores, using a Kernel (Epanechnikov) algorithm with a first-stage logit model. * *P* <0.05, ** *P* <0.01, *** *P* <0.001

Supplemental Table 9: Estimated PSM associations between birth in an intervention village and adult reproductive outcomes – radius matching

|  |  | Ages at menarche | Ages at first pregnancy | Whether had at least one child | Total number of children born |
| --- | --- | --- | --- | --- | --- |
|  | n | Estimate  (95% CI) | Estimate  (95% CI) | Estimate  (95% CI) | Estimate  (95% CI) |
| Index men and women | 1,358 | NA | NA | -0.05 (-0.09, 0)* | -0.1 (-0.18, -0.02)* |
| Index women | 518 | 0.45 (0.23, 0.68)*** | 0.54 (0.05, 1.03)* | -0.1 (-0.19, -0.01)* | -0.22 (-0.39, -0.05)* |
| Index men | 835 | NA | NA | -0.03 (-0.06, 0) | -0.05 (-0.09, 0)* |
| Married index men and married index women | 427 | NA | NA | -0.01 (-0.1, 0.09) | -0.09 (-0.27, 0.09) |
| Married index women | 326 | 0.32 (0.04, 0.6)** | 0.54 (0.05, 1.03)* | 0.01 (-0.09, 0.11) | -0.1 (-0.31, 0.11) |
| Married index men | 101 | NA | NA | -0.03 (-0.24, 0.18) | -0.1 (-0.39, 0.2) |

Note: Values are propensity score matching estimates of associations between birth in an intervention village and outcome variables, along with 95% confidence intervals (CI). Matching was on propensity scores, within a probability radius of 0.05. * *P* <0.05, ** *P* <0.01, *** *P* <0.001

Supplemental Table 10: Estimated PSM associations between birth in an intervention village and adult reproductive outcomes – one-to-one nearest-neighbor matching

|  |  | Ages at menarche | Ages at first pregnancy | Whether had at least one child | Total number of children born |
| --- | --- | --- | --- | --- | --- |
|  | n | Estimate  (95% CI) | Estimate  (95% CI) | Estimate  (95% CI) | Estimate  (95% CI) |
| Index men and women | 1,358 | NA | NA | -0.05 (-0.09, -0.01)* | -0.09 (-0.16, -0.02)** |
| Index women | 518 | 0.34 (0.12, 0.56)*** | 0.46 (-0.03, 0.94) | -0.06 (-0.15, 0.02) | -0.15 (-0.31, 0.01) |
| Index men | 835 | NA | NA | -0.02 (-0.05, 0.01) | -0.04 (-0.08, 0) |
| Married index men and married index women | 427 | NA | NA | 0.01 (-0.08, 0.1) | -0.05 (-0.23, 0.12) |
| Married index women | 326 | 0.33 (0.08, 0.58)** | 0.46 (-0.03, 0.94) | 0.01 (-0.09, 0.1) | -0.07 (-0.27, 0.13) |
| Married index men | 101 | NA | NA | -0.07 (-0.26, 0.13) | -0.07 (-0.35, 0.21) |

Note: Values are propensity score matching estimates of associations between birth in an intervention village and outcome variables, along with 95% confidence intervals (CI). Matching was on propensity scores, using a one-to-one nearest neighbor (without replacement) matching algorithm. * *P* <0.05, ** *P* <0.01, *** *P* <0.001
